# Postmarketing Safety Study Tool: A Web Based, Dynamic, and Interoperable System for Postmarketing Drug Surveillance Studies

**DOI:** 10.1155/2015/976272

**Published:** 2015-10-12

**Authors:** A. Anil Sinaci, Gokce B. Laleci Erturkmen, Suat Gonul, Mustafa Yuksel, Paolo Invernizzi, Bharat Thakrar, Anil Pacaci, H. Alper Cinar, Nihan Kesim Cicekli

**Affiliations:** ^1^SRDC Software Research & Development and Consultancy Ltd., ODTU Teknokent Silikon Blok No. 14, 06800 Ankara, Turkey; ^2^Department of Computer Engineering, Middle East Technical University, 06800 Ankara, Turkey; ^3^Lombardia Informatica S.p.A., Via Torquato Taramelli 26, 20124 Milano, Italy; ^4^F. Hoffmann-La Roche, 4070 Basel, Switzerland

## Abstract

Postmarketing drug surveillance is a crucial aspect of the clinical research activities in pharmacovigilance and pharmacoepidemiology. Successful utilization of available Electronic Health Record (EHR) data can complement and strengthen postmarketing safety studies. In terms of the secondary use of EHRs, access and analysis of patient data across different domains are a critical factor; we address this data interoperability problem between EHR systems and clinical research systems in this paper. We demonstrate that this problem can be solved in an upper level with the use of common data elements in a standardized fashion so that clinical researchers can work with different EHR systems independently of the underlying information model. Postmarketing Safety Study Tool lets the clinical researchers extract data from different EHR systems by designing data collection set schemas through common data elements. The tool interacts with a semantic metadata registry through IHE data element exchange profile. Postmarketing Safety Study Tool and its supporting components have been implemented and deployed on the central data warehouse of the Lombardy region, Italy, which contains anonymized records of about 16 million patients with over 10-year longitudinal data on average. Clinical researchers in Roche validate the tool with real life use cases.

## 1. Introduction

It is a well-accepted fact that, due to the limited scope and duration of clinical trials, drugs may still have serious side effects, adverse drug reactions (ADRs), after they are marketed. Postmarketing drug surveillance systems have been in place in order to analyze additional information about a drug's safety, efficacy, and optimal use to capture such ADRs. During the last decades, postmarketing activities in pharmacovigilance have largely depended on spontaneous case reports, which is still the case unfortunately. There are certain limitations on surveillance activities with spontaneous report data [1–4]. Postmarketing surveillance is also of vital importance for pharmacoepidemiology, especially for evidence development about effectiveness, safety, and quality of drugs in terms of ADRs [5, 6].

At present, postmarketing drug surveillance is largely being carried out with traditional methods for both pharmacovigilance and pharmacoepidemiology. In pharmacovigilance, there is active research on data mining algorithms [7] running on spontaneous report databases. On the other side, dedicated cohort and case-control studies are being performed within pharmacoepidemiological research. Although these traditional methods are currently dominant, a new research area that uses the already available electronic health data for clinical research purposes is emerging, which is referred to as the secondary use of Electronic Health Records (EHRs). EHRs provide a huge, but still underutilized, source of information on the real world use of drugs for observational studies. Although EHRs are primarily designed for patient care, they also contain a broad range of clinical information highly relevant to surveillance studies. EHR data available in clinical care systems can clearly complement and strengthen existing postmarketing safety studies [3, 8, 9]. Relative to spontaneous reports, EHRs cover extended parts of the underlying medical histories, include more complete information on potential risk factors, and are not restricted to patients who have experienced an adverse drug reaction.

Successful utilization of available EHRs for clinical research in terms of access, management, and analysis of patient data within and across different functional domains is a critical factor in terms of secondary reuse [9]. In line with this vision, there are important efforts for building large data pools from the EHRs to benefit from the available longitudinal observational data. The Sentinel Initiative of the U.S. Food and Drug Administration (FDA) aims to build a distributed network for active postmarketing surveillance for drug safety in the USA [10, 11]. The Observational Medical Outcomes Partnership (OMOP) is another important initiative targeting a similar objective for improvements in postmarket drug monitoring [12]. There are several other pharmacoepidemiological databases such as the Clinical Practice Research Datalink (CPRD) [13] which is based on the General Practice Research Database (GPRD) experience in the UK and The Health Improvement Network (THIN) database containing longitudinal medical data [14]. As a natural result, data mining on such national and international data pools appears as a new research area for signal detection and safety monitoring [3, 4].

The objective of the aforementioned initiatives is to use the available EHR data held by multiple different systems for clinical research purposes (mainly for postmarketing surveillance, comparative effectiveness research, and evidence development). In addition to the distributed architecture of the Sentinel Initiative, recent research projects like SALUS (Scalable, Standard based Interoperability Framework for Sustainable Proactive Post Market Safety Studies) [8], TRANSFoRm (Translational Research and Patient Safety in Europe) [15], and EHR4CR (Electronic Health Records for Clinical Research) [16] address the different levels of the interoperability problem between the clinical research and patient care domains with a distributed perspective.

Current research on postmarketing surveillance for pharmacovigilance and pharmacoepidemiology tries to unify the available EHR data on a common information model. Most of the time, this forces the EHR systems to implement the necessary adapters for transforming data into the defined common model and persist in a separate database. Either distributed or not, analyses on longitudinal EHR data require clinical researchers to implement the designed algorithms and build methods according to the predefined data model of the database that they are working on. On the other hand, some approaches transform the query to the native data model at each transaction. It is an experienced fact that data and processing requirements of different areas of clinical research change in time while the quality, quantity, and availability of EHR data on patient care side increase. In parallel with this, new initiatives propose new common data models into which collaborating EHR sources have to transform and transfer data, regardless of the system's nature. The literature exemplifies this situation clearly.

Vaccine Safety Datalink [17] is an early initiative for transforming EHR data for postmarketing safety surveillance of vaccines. FDA's Sentinel Initiative and the Mini-Sentinel pilot system [10, 11] are one of the latest and important efforts for postmarketing surveillance, built on the experiences of Vaccine Safety Datalink. Mini-Sentinel builds a distributed system to answer safety queries of clinical researchers through a common information model. OMOP [12] introduces its own common data model (CDM) to transform EHR data. Informatics for Integrating Biology and the Bedside (i2b2) [18] is another parallel effort with similar objectives that exposes its own common information model. CPRD [13] is a European example of the latest pharmacoepidemiological databases and there are several ongoing projects supported by European Medicines Agency and European Commission using a common information model for surveillance activities. The fact is that those common information models are not so “common”; they are only used within the boundaries of the associated initiatives and projects.

In this paper, we address the heterogeneity problem among common data models for clinical researchers who work on EHR data for postmarketing surveillance studies. We show that this problem of interoperability can be solved in an upper level with the use of common data element (CDE) phenomenon [19]. If the applications share the machine-processable definitions of the data elements and there are established links between data elements of different domains (i.e., clinical research and patient care domains), this can be used to facilitate automatic access to data across different domains. Hence, in the context of postmarketing surveillance, uniform observational analysis methods can be designed and implemented independently of the underlying data model, whether the source is a pharmacoepidemiological database or directly a hospital information system.

In the light of the common data element based interoperability approach, we design and implement the Postmarketing Safety Study Tool (PMSST) which can extract any needed information from a patient record after it is retrieved as a result of an eligibility query or it is directly accessed from the EHR database within a data mining routine. Our design is built upon the notion of CDEs and makes use of a Semantic Metadata Registry (MDR) to retrieve data element definitions and use their extraction specifications to access data [19, 20]. With the use of the extraction specifications, PMSST lets the researcher define what needs to be extracted from the patient records with the help of the abstract CDE definitions accessed from a semantic MDR [19]. With this dynamic behavior, the researcher writes her methods on the schema/template which will be created based on the data elements that she manipulates. With the help of the underlying interoperability framework [19], postmarketing surveillance methods do not have to be restricted to the data model of an EHR source.

## 2. Materials and Methods

The tool that we introduce in this paper has been built within the SALUS interoperability framework. Hence, first of all, SALUS project and its incorporated common data element based interoperability framework are introduced in Section 2.1. Afterwards, Section 2.2 outlines the general design principles of the PMSST on top of a use case scenario from the SALUS project. And Section 2.3 describes the implementation and finalizes the Materials and Methods.

### 2.1. SALUS Project

SALUS aims to create a semantic interoperability layer in order to enable the secondary use of EHR data for clinical research activities. SALUS follows a common data element based interoperability approach and uses the semantic MDR to maintain its common data elements (CDEs). Built upon its abstract CDE definitions, SALUS exposes a semantic RDF [21] based content model as its common information model. SALUS project deals with different content models both in clinical care (i.e., HL7/ASTM CCD [22] and CEN/ISO 13606 [23]) and clinical research domains (i.e., SDTM [24] and OMOP CDM [12]) and harmonizes them in the SALUS common information model (CIM) [25]. Through its semantic interoperability layer, SALUS accepts eligibility queries and returns resultant patient summaries as instances of SALUS CIM.

Several organizations are publishing common data element dictionaries and common models in order to solve the interoperability problem within and between clinical research and patient care borders. The objective is to provide a dictionary like the collection of the abstract definitions of common data elements. Most of the time, these definitions are published as unstructured text files. Rarely, semistructured spreadsheets are used to publish the data element specifications. Health Information Technology Standards Panel (HITSP) is one of such organizations publishing a library of common data elements, called HITSP C154: Data Dictionary [26]. One CDE from this dictionary is “Conditions Problem Code” which is defined as the code describing the medical problem according to a specific vocabulary of problems. This abstract CDE definition can be bound to an implementation such as HL7/ASTM CCD with an XPath script as in the following snippet:

 
/cda:ClinicalDocument/cda:component/cda:
 
structuredBody/cda:component/cda:section
 
/cda:entry/cda:act[cda:templateId/@root
 
=＇2.16.840.1.113883.10.20.1.27＇]/cda:
 
entryRelationship[@typeCode=＇SUBJ＇]/cda:
 
observation[cda:templateId/@root=＇2.16.
 
840.1.113883.10.20.1.28＇]/cda:value
 
/@code

Data retrieval mechanism of the SALUS enabled clinical research tools has been built on top of the idea of data interoperability through federated semantic metadata registries [27] where the machine-processable CDE definitions play a crucial role. The CDE based data interoperability approach introduces a federated system in which the abstract and machine-processable data element definitions in ISO/IEC 11179 [28] formalism are managed within metadata registries and can be linked/mapped with each other by using semantic web technologies. This approach makes a clear distinction between the abstract definitions and implementation dependent parts of the data elements.

The abstract SALUS common data element (CDE) [29] definitions published in the scope of SALUS project are maintained within a semantic MDR. Each CDE has its extraction specification which can be executed on SALUS CIM conformant patient data to extract the indicated piece of information. The extraction specifications of the SALUS CDEs are SPARQL [30] scripts since SALUS common information model (CIM) is RDF based.

**Figure 1:**
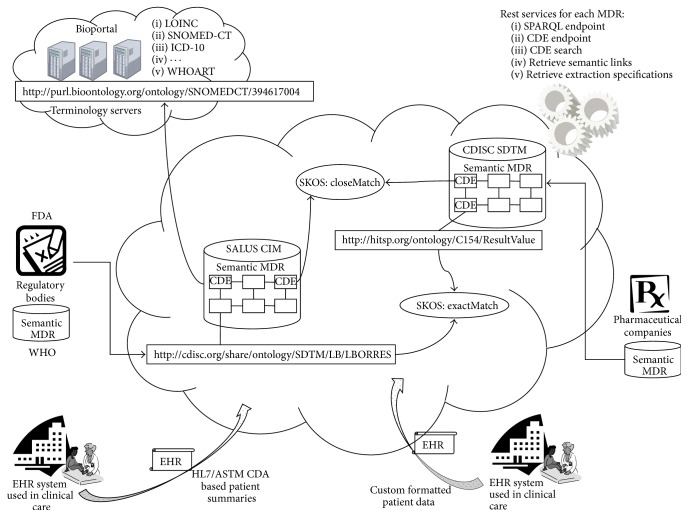
CDE based data interoperability framework through federated semantic metadata registries.

As illustrated in Figure 1, different organizations or standardization bodies can maintain their own CDE specifications inside the semantic MDR architecture and establish the semantic links to other CDEs from other systems or domains. If one abstract CDE set has the access method (so called extraction specifications such as an XPath or SPARQL script) to an implementation specific content model (i.e., XML based HL7/ASTM CCD or RDF based SALUS CIM), the semantic link chain among the CDEs can be traversed in line with the linked data principles and data can be extracted from the instance conforming to that content model pointed by the extraction specification. Compared to static message translation between different specifications, this approach well integrates with the distributed architectures and does not need to perform costly operations such as parsing and message construction. The semantic MDR provides the necessary functionality together with the interfaces so that the users and semantic-aware applications can interact with the system easily.

PMSST is one of the safety analysis tools developed within the scope of the SALUS project. Within this CDE based data interoperability framework, PMSST retrieves the CDE definitions from a semantic MDR where any common data element model can be maintained according to ISO/IEC 11179 metamodel [28]. Study Data Tabulation Model (SDTM) is a standard data model for the pharmaceutical companies while submitting information about clinical studies to FDA. Pharmaceutical companies like Roche use SDTM variables for data annotation during their postmarketing surveillance studies. In our implementation, the registry maintains the SDTM variables and the SALUS common data elements [29], and there are semantic links between SDTM and SALUS data elements as introduced by Sinaci and Laleci Erturkmen [19] and as illustrated in Figure 1.

Using PMSST, a clinical researcher designs a data schema (a template) by using SDTM variables on which she writes scripts (i.e., SAS [31]) for surveillance studies. The system knows how to extract information from the underlying EHR data by using the extraction specifications of the CDEs. Therefore, the researcher is not bound to the data model of the underlying database; it could be a system providing HL7/ASTM CCD [22] based patient summaries or an OMOP database or any other EHR database as well as a pharmacoepidemiological database. As long as the appropriate extraction specifications (i.e., XPath [32] scripts for HL7 CCD) are available, PMSST can extract the necessary information. The communication with the metadata registry is carried out through an international interoperability profile, namely, IHE Data Element Exchange (DEX) profile [20] in which PMSST implements the metadata consumer role while the semantic MDR implements the metadata source role.

### 2.2. Design Considerations

Postmarketing Safety Study Tool is a web based tool enabling clinical researchers to extract data from different EHR systems by designing data collection sets through common data elements. After patient record is retrieved as a result of an eligibility query, any needed information can be extracted from the patient record to populate the data collection set with the help of abstract CDE definitions used to annotate data collection set definitions. By means of the underlying interoperability framework [19], PMSST enables researchers to develop analysis method on the data collection set schema defined by abstract CDEs without being concerned about the structure of the underlying data source. The driving force during the design phase of PMSST is a real world use case identified by the pharmaceutical company Roche in the scope of SALUS project.

#### 2.2.1. PMSST Use Case

*Background of the Use Case.* Congestive heart failure (CHF) is a leading cause of hospitalization for patients aged 65 years and older. CHF is of particular concern in diabetic patients in whom incidence rates are two to five times greater than those in the general population. The United Kingdom Prospective Diabetes Study (UKPDS) estimated incidence rates of 2.3–11.9 cases per 1000 patient-years in diabetic patients. Several risk factors of CHF in diabetic patients have been identified. These include, for example, duration of diabetes, history of ischemic heart disease, renal function, hypertension, diabetes treatments, and HbA1C. However, the incidence of CHF in diabetic patients with a recent acute coronary event is not fully known. In particular, no estimates of CHF for different treatment regimens are available in these patients.

Roche is conducting clinical trials in both acute coronary syndrome (ACS) patients and in ACS patients with diabetes. Whilst the trials are blind, it is important to compare the observed overall incidence rate of an important adverse event like CHF in the trials with that in similar background populations. Such a comparison provides a context to the observed incidence and enables us to identify any potential safety concerns earlier on (e.g., if the observed incidence in the trial is greater than the background). 

*Objective.* The objective of this use case is to estimate incidence rates of CHF in diabetic patients with a recent acute coronary syndrome (ACS) event considering other diabetic medications of patients such as type 2 diabetes (T2D) and related treatment regimens as well. The estimation results should be stratified based on patient demographics such as age or gender. 

*Patient Selection.* Identify all patients with a first ACS event defined by acute myocardial infarction or unstable angina during the period 2005 to 2011. Include only those patients who have a minimum of 1-year history prior to the ACS. Exclude those patients who died within 30 days after the ACS event. Exclude those patients aged less than 18 at the time of ACS. The remaining patients define an ACS cohort of interest. For each patient, the STARTDATE is set to 30 days after the ACS event (so if a patient has an ACS on 5th July 2007, his start date is set to 4th August 2007). We allow a 30-day delay to ensure the ACS has stabilized. For each patient define the LASTDATE as the minimum of date of death or the date the patient transfers out of the system and can provide no more data, that is, 31st Dec 2011.

*Data Collection Set Definition.* For the ACS cohort described in the previous section, we identified the necessary data that needs to be gathered as a data collection schema (like a common information model required for this surveillance study) composed of several schema items which can be resembled to the columns of a relational database table. While some of the data collection set schema items can be extracted from the EHR data using the extraction specification of a single SDTM data element, some of them require further calculations. For instance, whilst the start date of the ACS event can be extracted in a single operation, we should take the start date of the ACS event into account to be able to produce the result for “average systolic blood pressure (BP) over 12 months before the start of ACS” schema item; it requires querying of particular measurements within a particular timeframe and calculation of the mean value.

Table 1 shows a sample of the schema items together with the corresponding SDTM data elements. In order to calculate the final results for the schema items, some of the data elements should be provided with specific MedDRA [33] codes to indicate the values according to the requested information which are also indicated in Table 1. For example, “Date of Acute Myocardial Infarction” is described with MH.MHPTCD = 10000891, MHSTDTC = ?.

#### 2.2.2. How the Use Case Affected PMSST Design?

Patient selection phase is the execution of the eligibility criteria for retrieving the data of the defined cohort. For PMSST, this execution is handled through the semantic interoperability layer of SALUS. However, this could be any other system like Sentinel or Query Health [34] from which data is retrieved in the form of a content model. The data collection set schema is defined by annotating the schema items with the CDEs that are already being used in research community, in our case SDTM data elements. As long as the extraction specifications of the selected CDEs to that content model are available from the EHR data sources and reachable with appropriate links in the semantic MDR, PMSST can perform the same execution to build data according to the schema defined by the researcher with the help of the CDEs.

Analyzing the use case, we elicited the key requirements for PMSST and based the design of the key functionalities of PMSST on these requirements. During the data collection set schema definition process, values of particular schema items might be used in defining other schema items. Therefore, PMSST provides a flexible variable definition mechanism. PMSST keeps track of the variable definitions and generates the queries to be applied on the EHR data and organizes their execution order.

As it can be seen in Table 1, some of the schema items need further calculation such as average value of blood pressure measurements, date of the first occurrence of T2D diagnosis, and last weight value before the ACS event. We design PMSST such that it would present different selection and calculation options automatically considering the value domain of the schema item.

Value domains of the used CDEs may be referring to different terminology/coding systems. For example, while asking whether a patient has T2D or not, researcher at Roche uses MHPTCD common data element from the MH domain of SDTM. Since this data element requires a coded value from MedDRA, the researcher should easily assign values to such data elements during her schema design. For this purpose, PMSST has been integrated with a terminology server so that it would recommend possible values based on the schema item through a type-a-head search mechanism.

### 2.3. System Description

PMSST is a web based tool which can be used via modern web browsers. It has been implemented with the latest high performance web technologies incorporating HTML5 design principles and RESTful client-server communication. The tool is composed of an eligibility query execution and a data selection part. Details of the former are out of the scope of this paper. Upon the execution of an eligibility query, a cohort of patient data is retrieved in the form of a content model adopted by the EHR sources. We claim that the CDE based interoperability implementation of PMSST can make use of any content model as long as the appropriate extraction specifications are available for the abstract CDE definitions within the semantic MDR framework.

**Figure 2:**
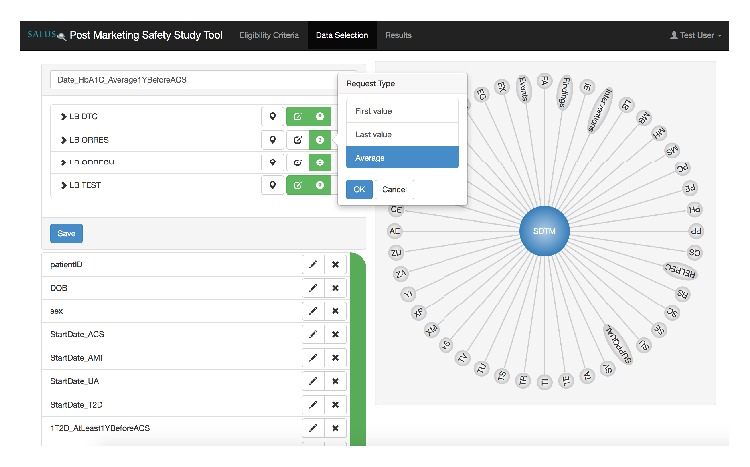
A snapshot of PMSST while the researcher defines a data collection set schema. On the right hand side, domains of SDTM form a circle; if selected, then CDEs of that domain form the circle. On the left hand side, a schema item “Date_HbA1C_Average1YBeforeACS” is created out of 4 SDTM elements. Below that, a list of other schema items is shown.

Figure 2 presents the data selection phase of PMSST. The user can define a data collection set schema at this phase by using the CDE definitions retrieved from the semantic MDR. In our implementation, the registry maintains SDTM variables and SALUS CDE set according to ISO/IEC 11179 metamodel principles. When the user decides to use SDTM, the object classes (aka domains) are presented to the user to give a top-down browsing experience. When a domain is selected, the data elements created out of that object class are presented to the user. When a CDE is selected, it appears on the left hand side to create further calculations. Once a schema item is designed, it is saved and the overall schema design continues. The user can edit or delete an existing item anytime during the design phase.

#### 2.3.1. Data Flow between Components

**Figure 3:**
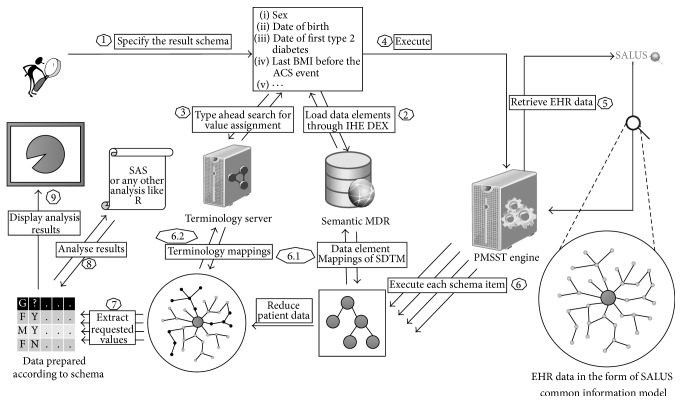
Step-by-step representation of the data flow between different components. A clinical researcher uses PMSST in order to define a data collection set schema so that when patient data is retrieved from the underlying EHR source(s), data will be automatically transformed to that schema.

PMSST is composed of several different components among which a number of integration mechanisms exist. In Figure 3 the flow of data between those integrated components is depicted and the steps of the flow are described in as follows.

1. The researcher uses a web browser to define the data collection set schema by using the CDEs. Roche researchers use SDTM variables in our deployment as identified in Table 1.
2. CDEs are maintained in the semantic MDR and retrieved through the IHE DEX profile. The user browses the CDEs starting from the object classes in a top-down fashion.
3. If the user likes to restrict the value of a selected data element (i.e., set acute myocardial infarction to MHPTCD element), possible values can automatically be searched from the terminology server. PMSST knows in which coding system to look for the term by analyzing the value domain of the CDE definition automatically.
4. After the user completes the schema definition, identifying each schema item by using abstract CDE definitions, the schema definition is sent to the PMSST engine on the server side.
5. Eligibility query is sent to the SALUS system and EHR data of the eligible patients is retrieved in the form of SALUS common information model.
6. For each schema item definition, PMSST engine extracts information from the EHR data and performs necessary calculations to place into the appropriate location according to the schema definition.
  (6.1) Schema is defined by SDTM elements. Semantic MDR keeps the mappings between SDTM and SALUS CDEs as presented in Table 2 in the next section, and SALUS CDEs have the extraction specifications to access the necessary information from the EHR data. CDE definitions, mappings, and extraction specifications are retrieved from the semantic MDR in conformance to the IHE DEX profile. Since SALUS CIM is an RDF based model, the extraction specifications of the SALUS CDEs are SPARQL scripts.
  (6.2) If the schema item definition includes a value in one of its defining CDEs, value analysis should be done. However, in our deployment, EHR data is coded with ICD-9-CM terminology system for patient conditions while SDTM elements refer to MedDRA or NCI terms. The terminology server includes mappings between these different coding systems, and PMSST can do value matching with the help of this terminology server.
7. As a result of these data extraction operations, the data collection set is populated conforming to the schema defined by the researcher.
8. User can write analysis methods on top of this schema independently of the underlying EHR source model. In our deployment, Roche implements SAS scripts to do further analysis.
9. Finally the analysis results are presented to the researcher.

#### 2.3.2. How Semantic Interoperability Is Achieved through the Use of CDEs and a Semantic MDR

The CDE based data interoperability approach lets the PMSST interact with the semantic MDR through IHE DEX profile and retrieve abstract CDE definitions. The researcher interacting with the PMSST uses the data elements that she is used to in her research domain. The underlying architecture of the PMSST does not make any message translation between different content models (i.e., from SALUS CIM based patient summaries to SDTM conformant instances). Instead, the abstract CDE definitions and their semantic links maintained by the semantic MDR are processed in order to find an extraction specification to be executed on the content model to which the EHR data conforms. This clear distinction between the abstract and implementation dependent parts of the CDEs enables integrating the CDEs with the semantic web technologies and linked data principles by using the semantic MDR.

In our semantic MDR, the links between different sets of abstract CDE definitions can be established through well-known knowledge organization systems such as SKOS (i.e., skos:exactMatch) or any property can be indicated again with SKOS (i.e., skos:notation) or other ontological constructs.

PMSST makes use of the abstract CDE definitions of the SDTM variables retrieved from the semantic MDR. In order to enable the retrieval of the extraction specifications given the SDTM variables, we mapped the SDTM elements to the SALUS CDEs. We implemented an automatic content model importer on top of the open API of the semantic MDR for importing the SDTM variables and their mappings to SALUS CDEs. In this way, although the user defined the data collection set via SDTM variables, he becomes able to collect the requested data from the EHRs sources that can provide the EHR data of the eligible patients through SALUS common information model.

In the semantic MDR, SALUS CDEs have also mappings to HITSP C154 Data Dictionary [26] elements through skos:exactMatch semantic links. Semantic MDR is capable of processing these semantic links to establish the links between SDTM variables and HITSP C154 elements transitively. Table 2 lists some part of the mappings used during the execution of our implementation.

Although the usage of the tool starts with defining eligibility criteria and retrieving EHR data according to that query, our implementation is independent of the content model according to which the EHR data is shaped. For example, if the underlying EHR system can provide HL7/ASTM CCD based patient summaries, then PMSST can seamlessly process the data by using the corresponding extraction specifications retrieved from the semantic MDR. Because HITSP C154 defines XPath expressions from its CDE definitions to HL7/ASTM CCD based documents and PMSST can retrieve the extraction specifications through the HITSP C154 mappings, this time, the extraction specifications would be XPath expressions and clinical researcher would not be aware of this. This means that PMSST can automatically communicate with an EHR system which is capable of exporting HL7/ASTM CCD based document summaries and make the data available for clinical research automatically.

## 3. Results and Discussion

In the context of the SALUS project, PMSST and all related components have been implemented and deployed on top of the SALUS interoperability framework integrated with the central data warehouse of the Lombardy region, Italy. This regional database includes anonymized data of ~16 million patients with over 10-year longitudinal data on the average. Clinical researchers in Roche are validating the PMSST with real life use cases, one of which is presented in this paper. Till the actual deployment within SALUS, we worked with simulated data to collect further requirements from clinical researchers and improve the capabilities of PMSST.

For the eligibility criteria defined in the use case introduced in this paper, PMSST retrieved anonymized data of ~8000 acute coronary syndrome (ACS) patients from a population of ~16 million patients. The definition of the data collection set schema starts after retrieving the cohort based on the eligibility criteria. PMSST transforms the retrieved cohort data to the model defined by the data collection set schema. The eligibility query execution is not in scope of this paper.

The researchers in Roche cannot directly see the resultant data according to the privacy rules of the SALUS project. Instead, their analysis methods are executed on the returned dataset and the results of this execution are presented through the graphical user interface of PMSST. Researchers from Roche have implemented SAS scripts assuming that in the end they will have the data represented with SDTM variables, which they already use in their daily work. However, the data warehouse of the Lombardy region has a custom schema. SALUS technical and semantic interoperability solutions retrieve data from this custom database and transform to instances of SALUS common information model (CIM). The CDE based data interoperability approach has enabled the mappings between the SDTM variables and SALUS CDEs where the SALUS CDEs have their extraction specifications (SPARQL scripts in this case). With the help of this architecture, PMSST can extract data from the SALUS CIM based patient data by using the SDTM variables. Afterwards, the analysis routines (i.e., SAS scripts) of the clinical researchers run on the SDTM conformant data.

In order to assess the validity of the data collection set calculated by PMSST, we have conducted a comparative analysis. By issuing SQL queries to the data warehouse of the Lombardy region, we have obtained several statistics regarding the items in the data collection set and compared them with the set populated by PMSST. For instance, demographic analysis on Lombardy region data warehouse shows that 38.22% of ACS patients are females which is equal to the percentage of “Female” in “Sex” column of the resultant PMSST data collection set. Similarly, PMMST calculates the incidence “Patient died any time after start of ACS” as 34.63% which aligns with the real values calculated in LISPA data warehouse. These analyses over the items of the data collection set show that the dataset created by PMSST is correct compared to the original data and assure us about the reliability of the results.

One problem we observed during the analysis is that many items of data collection set defined in Roche use case were actually empty. For instance, there was no data in any of the patients regarding the systolic and diastolic blood pressure measurements, or history of smoking. To deduce the cause of the problem, we have investigated the LISPA data warehouse and found out that the available data is not fully structured. Those empty columns in the resultant data collection set were also missing in the data warehouse. This has hindered Roche researchers from full utilization of the PMSST according to the planned use case. On the other hand, the records which exist in the data warehouse, such as the ones related to congestive heart failure (CHF), have been processed by the tool accurately. Thus, we conclude that PMSST can be fully exploited once the underlying EHR source gets more structured and includes more data about the patients.

### 3.1. End-User Validation

In order to assess whether PMSST fulfills the intended use from an end-user point of view or not, it has been tested and evaluated by real end-users from Roche in the scope of the SALUS project. An evaluation and validation framework based on the ISO/IEC 25040 Software engineering, Software Product Quality Requirements and Evaluation (SQuaRE) standard has been developed. According to the developed framework, a total of 6 users including a data analyst and an epidemiologist have taken part in the evaluation in order to assess the feasibility of conducting a study over a particular EHR system by using PMSST.

We have built a PMSST specific questionnaire which is based on the Health IT Usability Evaluation Scale [35] and provided to the evaluators online. The results obtained from the questionnaire based evaluation can be summarized as follows:

- PMSST has been a positive addition to postmarketing safety studies.
- Using PMSST makes it easier to define data fields (the Data Selection tab) to be extracted from the retrieved patient summaries.
- Using PMSST enables defining data collection fields and performing data selection more quickly.

Apart from the questionnaire results, the evaluators provided specific comments addressing the benefits of PMSST in their studies. Currently Roche conducts safety analysis studies based on some sample EHR datasets it has. It has been concluded that having a tool like PMSST which enables extraction of selected data collection sets of a specified cohort selection from different EHR systems would be very beneficial for pharmaceutical companies as it will increase the size and variability of patient data pools. The data analyst and the epidemiologist from Roche positively agree on that PMSST has been successful to achieve its focused objective and the data provided by the tool is feasible and suitable for a wider range of observational clinical studies. On the other hand, they also report that the efficiency of the tool and the completeness of the data vary depending on the status of selected EHR data source.

### 3.2. Current Status and Availability of Software

The deployment in Lombardy region, Italy, can only serve the SALUS project partners (i.e., Roche) behind high security firewalls because of the privacy concerns. For the interested readers, we prepared a dataset of 50 simulated patients considering the real world facts that Roche has experienced from previous studies and ongoing work on Lombardy region database. A package of the deployable software including this simulated dataset can be requested from the corresponding author.

## 4. Conclusions

The PMSST, introduced in this paper, enables clinical researches to define data collection set schemas on which the postmarketing safety studies will be conducted without being concerned about the structure of the underlying data sources. The main benefit of utilization of CDE based interoperability architecture is the ability of developing surveillance methods which do not have to be restricted to the data model of the EHR source: cohort selection and data collection set definition can be easily done in researchers' own language (such as SDTM). Moreover, such an interoperability architecture allows the data collection operation to be run on distributed EHRs resources which might be using different content models to expose patient data.

## Figures and Tables

**Table 1:** Data collection set schema details for the PMSST use case.

Scheme item description	Data Elements of the Schema Item	Corresponding SDTM data element name	MedDRA code for MH.MHPTCD

Sex	Sex	DM.SEX	

Date of acute Coronary syndrome (ACS) event	(i) ACS event (ii) Start date of ACS event	(i) MH.MHPTCD (ii) MH.MHSTDTC	10051592

Date of acute myocardial infarction	(i) Acute myocardial infarction (ii) Start date of acute myocardial infarction	(i) MH.MHPTCD (ii) MH.MHSTDTC	10000891

Date of unstable angina	(i) Unstable angina pectoris (ii) Start date of unstable angina pectoris	(i) MH.MHPTCD (ii) MH.MHSTDTC	10002388

Had a congestive heart failure (CHF) before start of ACS (Y/N)	(i) Congestive heart failure (ii) Congestive heart failure time indicator	(i) MH.MHPTCD (ii) MH.MHSTDTC	10007559

Had a CHF after start of ACS (Y/N)	(i) Congestive heart failure (ii) Congestive heart failure time indicator	(i) MH.MHPTCD (ii) MH.MHSTDTC	10007559

**Table 2:** Mappings of the common data elements: SDTM, SALUS CDE set, and HITSP C154 Data Dictionary.

SDTM	SALUS CDE	HITSP C154
DM	Patient	Personal Information
DM.DMSEX	Patient.Gender.CD	1.06 Personal Information Gender
MH	Patient.Condition.Condition	Conditions
MH.MHPTCD	Condition.ProblemCode.CD	7.04 Conditions Problem Code
MH.MHSTDTC	Condition.TimeInterval.IVLTS	7.01 Conditions Problem Date
